# Sanitary Legislation

**Published:** 1859-01

**Authors:** 


### SANITARY LEGISLATION.

There is not much to be said in this department on the present occasion, for the parliamentarians have been taking their ease, and active sanitary legislation of all kinds is meanwhile suspended.

An outbreak of *Endemic Fever at Windsor* has led to some discussion, and Mr. Simon and Mr. Austin have made inquiries as to cause, and found the cause to be local.

The gigantic scheme for the *Main Drainage of London,* adopted by the Metropolitan Board of Works, proceeds showly, but much too fast, after all. The plan being determined on, it is not necessary to do more now than regret. The fact is, the Metropolitan Board have been driven into the acceptance of this scheme by popular cry that something must be done.

Mr. Neison, the actuary, has opposed the theory of the commissioners appointed to inquire into the *Sanitary State of the Army* regarding the production of phthisis pulmonalis by overcrowding. Mr. Neison's statistical inquiries go on their part to show "that deaths from consumption in overcrowded districts are increased in a much less ratio than the deaths from other causes." It were well if Mr. Neison would study physic as well as figures, for his argument, though alarming in its statistical details, has, running through it, an entire misapprehension of the whole question of phthisis and its causes.

The *Vaccination System* is, we hear, about to be reconsidered in Parliament. The present act is an entire failure, and the Registrars express their entire inability to carry out the rules imposed by it. The Registrar of a large metropolitan district informs us that vaccination returns are not made on even one-third of the children registered by him. *Apropos* to this remark, we may mention that in America, not less than in England, the vaccination question, as a State question, is beset with difficulties, and that vaccination is very imperfectly performed throughout the whole of the United States. At this time, a committee appointed by the Senate of the States is holding its meetings with a view to suggest an effective measure for the regulation and improvement of the sanitary police of New York. One point thus brought before the committee refers specially to vaccination and to the clauses of a proposed bill in which great power is given to the health officers of interfering with families affected with small-pox. The truly English cry of "freedom of the subject" is as loud in America as here, and we shall be interested in hearing how our cousins meet the difficulty of compounding between life and liberty.

